# S4-1 Learning from national initiatives from across the EU in HEPA policy monitoring and evaluation

**DOI:** 10.1093/eurpub/ckad133.018

**Published:** 2023-09-11

**Authors:** Chair(s):, Diane Crone, Anne Vuillemin

**Affiliations:** Cardiff Metropolitan University; Université Côte d'Azur

## Abstract

The monitoring and evaluation of HEPA policy are essential steps towards developing the evidence base to understand the process and impact of policy implementation as well as guide future policy design. The policy development and implementation cycle can be strengthened through effective monitoring and evaluation tools, as well as multi-sectoral engagement. The promotion of physical activity often requires multiple sectors such as public health, sport, urban design etc., and working in diverse settings.

In recent years, researchers and international organizations, such as WHO, have produced useful resources and guidance for monitoring and evaluating the impact of policy on a national level. The fast-growing interest in physical activity policy by national governments, for the purposes of increasing health and wellbeing can provide new opportunities for capturing the impact of HEPA policy and learning from other countries. The lack of available evidence on the process and effectiveness of HEPA policy remains a challenge when developing new policy. This highlights the need for sharing good practice from diverse policy contexts and HEPA settings.

The symposium presentations will discuss policy monitoring and evaluation tools and case studies to investigate the different implementation aspects of national HEPA policy in four EU countries: Germany, Ireland, Slovenia and Wales. The goal of this symposium is to share lessons from the development of instruments used in (physical activity) policy monitoring and evaluation and to present real-world case studies of cross-sector collaboration. The high quality and diversity of the tools and projects presented in the four presentations, makes this symposium beneficial to both novice and experienced researchers, policy-makers and practitioners. The attendees can update their knowledge regarding different methods and instruments used in physical activity policy monitoring and evaluation and learn from engaging multiple sectors in HEPA such as urban design, sport, environment and public health.

Reflections from this symposium will inform and enhance HEPA policy development and implementation in real-time and stimulate future research and practice in this field.

